# Inter-limb asymmetry of kinetic and electromyographic during walking in patients with chronic ankle instability

**DOI:** 10.1038/s41598-022-07975-x

**Published:** 2022-03-10

**Authors:** Hossein Tajdini, Zahed Mantashloo, Abbey C. Thomas, Amir Letafatkar, Giacomo Rossettini

**Affiliations:** 1 Biomechanics and Corrective Exercise Laboratory, Department of Biomechanics and Sport Injury, Faculty of Physical Education and Sports Sciences, Kharazmi University, Mirdamad Blvd., Hesari St, Tehran, Iran; 2 Department of Kinesiology, University of North Carolina at Charlotte, Charlotte, NC USA; 3 School of Physiotherapy, University of Verona, Verona, Italy

**Keywords:** Health care, Medical research, Nanoscience and technology

## Abstract

After an initial ankle sprain, a relevant number of participants develop chronic ankle instability (CAI). Compensatory strategies in patients with CAI may change the inter-limb symmetry needed for absorbing movement-related forces. Accordingly, an increased risk of injury can occur. The present study aimed to compare the inter-limb asymmetry of kinetic and electromyography between individuals with CAI and without a history of an ankle sprain (Non-CAI) during walking. In this cross-sectional study, fifty-six athletes (28 CAI; 28 Non-CAI) participated. Participants walked at a comfortable pace over level ground while vertical ground reaction force (vGRF) and muscle activity of the tibialis anterior, peroneus longus, medial gastrocnemius, and gluteus medius were recorded. Inter-limb asymmetry during walking was calculated for each of the variables. Patients with CAI exhibited a greater inter-limb asymmetry of the first peak of vGRF, time to peak vGRF, and loading rate (P < 0.001), as well as presenting a greater inter-limb asymmetry of peroneus longus activity (contact phase) (P = 0.003) and gluteus medius activity (midstance/propulsion phase) (P = 0.010) compared to the Non-CAI group. No other differences in vGRF or muscles activity were observed between the groups. Our findings indicate that patients with CAI walk with greater inter-limb asymmetry in vGRF and muscle activity in different phases of the gait cycle compared to Non-CAI group. Our results could inform future studies on gait training aimed to reduce asymmetry during walking in patients with CAI.

## Introduction

The ankle joint is a primary sublocation for injury in athletics^1,2^. Lateral ankle sprain, in particular, is a common musculoskeletal injury that is often found among adolescent athletes^3^. After an initial ankle sprain, recurrent sprains, subjective feelings of instability, and disabling symptoms such as pain and swelling continue to affect 40% of patients at least one-year post-injury^4^. Persistent “giving way” episodes and recurrent injury are the obvious features of a heterogeneous condition known as chronic ankle instability (CAI)^5^. Residual and persistent symptoms associated with CAI can lead to decreased physical activity^6,7^, reduced quality of life^8^, and an increased risk of ankle osteoarthritis after injury^9^. Both lateral ankle sprains and CAI have been linked to decreased cartilage health and post-traumatic osteoarthritis^10^, accounting for 78% of end-stage ankle osteoarthritis cases^10^.

Initial lateral ankle sprains and subsequent sprains (e.g., CAI) damage the sensorimotor system and can alter the dynamic restraint mechanisms of the lower extremity^11^, impairing the ability to minimize joint loading and protect the joint against impairments^12^. Increased plantar flexion moment and impact ground reaction force in patients with CAI during initial contact of walking increase the load at the ankle joint in the sagittal plane^13^. It has been hypothesized that alterations in reflexive, sensory, and motor control strategies contribute to CAI^5^. For example, during walking, patients with CAI demonstrate strategies of neuromuscular-activation across their lower extremities, which were different from strategies presented by those Non-CAI^13–16^. However, the muscle activity profiles of patients with CAI during walking are conflicting. For example, patients with CAI have been reported to have increased peroneus longus and tibialis anterior activation during the early stance of walking^17^. Similar results have been reported by Delahunt et al.^18^ and Louwerens et al.^19^ concerning increased peroneus longus and tibialis anterior activation, respectively. On the other hand, some other studies^20–22^ have reported a lack of difference between CAI and Non-CAI groups. Further, research has also reported a significant decrease of tibialis anterior, peroneus longus, and gluteus medius activity during walking of patients with CAI^13^. It should be noted that these studies used different reference tasks for EMG normalization^13,18–22^, which complicates their direct comparison.

Patients with CAI have presented altered kinetics during walking^15^ and more intense physical activities such as running^23^. Specifically, patients with CAI had higher vertical ground reaction force (vGRF)^13,24,25^ and vGRF loading rates^24^, relative to Non-CAI individuals during early stance of walking. The vGRF and vGRF loading rate play a role in cartilage health^26–28^. Higher loading rates cause more chondrocyte death and cartilage matrix damage^27,28^. Patients with CAI experience changes in matrix densities as early as one year following initial injury^29^ and are at increased risk for joint degeneration and post-traumatic osteoarthritis^30^, indicating less cartilage resiliency and elasticity against repetitive load^31^. The relationship between altered loads and ankle articular cartilage health has not been assessed in patients with CAI up to now. However, the alteration of walking biomechanics (higher vGRF^13,24,25^, altered neuromuscular activation^14,15^, and altered joint kinematics at the ankle (e.g., increased inversion and reduced dorsiflexion)^13,18,32^) observed in patients with CAI might affect articular cartilage metabolism and health, thus increasing mechanical stress on a specific portion of the ankle cartilage. For example, Hintermann et al.^33^ showed that patients with CAI have 62% of ankle cartilage lesions in the talus's medial areas. It has also been reported that degenerative arthritis of the ankle has mainly occurred due to unbalanced mechanical loading in the medial ankle joint of CAI patients^34^. Therefore, quantification of vGRF differences in individuals with CAI compared to Non-CAI is warranted as a precursor to assessing the relationship between load and cartilage health in this population. Understanding differences in how patients with CAI load their ankles may assist with developing the treatment to slow ankle joint degeneration.

Frequently, ankle injuries cause bilateral defects, as patients compensate to protect the injured joint^35^, which increases the risk of contralateral limb injury^36,37^. Eighty-five percent of people who experience CAI after ankle sprains also have problems with the contralateral limb^38^. This inefficiency may contribute to the high recurrence rates in patients with CAI^38^. One possible way that can help to understand the mechanism of limb coordination in walking is to study inter-limb asymmetry. Investigating the asymmetry in patients with CAI can provide insights into the control of walking and the importance of bilateral deficits in the occurrence of injury. This finding may be unique from more conventional measures such as velocity and may guide the clinicians’ treatment decisions. Previous research emphasises optimal balance in lower limb symmetry of healthy individuals^39^. However, in limb-synchronous movement tasks such as walking, compensatory strategies may disturb the inter-limb symmetry necessary for absorbing movement-related forces, potentially increasing the risk of injury^40^.

There is limited research evaluating the compensatory strategies utilized by the sensorimotor system in patients with CAI, and the effect these may have on inter-limb symmetry. Additionally, previous research has reported mixed results regarding vGRF^24,25,41^ and muscle activity^14–16^ during walking in patients with CAI. Also, previous studies have not collected information about uninjured limb for CAI patients. Patients with CAI could exhibit biomechanical compensatory strategies to the contralateral limb but cannot be observed^42,43^. However, evaluating inter-limb asymmetry of vGRF and muscles activity in patients with CAI is essential, which can provide more complete information in these patients. Many of the features can be obtained from GRF and muscle activity to distinguish between normal and abnormal patterns of motor behavior. Thus, the study of the symmetry of GRF and electromyography variables in patients with CAI represents an opportunity because it is a less investigated area. Therefore, the present study aimed to compare the inter-limb asymmetry of vGRF components and selected lower limb muscles activity between individuals with CAI and Non-CAI during walking. We hypothesized that patients with CAI will exhibit greater inter-limb asymmetry in vGRF components and muscles activity compared with individuals without CAI during walking.

## Methods

### Study design and ethics

This study incorporated a single-session cross-sectional design that was conducted at the Biomechanics Laboratory of Kharazmi University. The independent variable was group (CAI, Non-CAI). The dependent variables were inter-limb asymmetry in vGRF components (first and second peak of the vGRF, vGRF loading rate, and time to peak vGRF) and muscle activity (tibialis anterior, peroneus longus, medial gastrocnemius, and gluteus medius). Prior to participation in the study, all subjects were briefed about the objectives and provided written informed consent, and all participants provided written informed consent prior to enrollment. This study was performed following the 1964 Helsinki declaration, its later amendments and local ethics committee by the Research Ethics Committee of the Sport Science Research Institute (IR.SSRI.REC.1400.1160).

### Inclusion and exclusion criteria’s

Eligibility criteria for the CAI group were based on the standards approved by the International Ankle Consortium^44^. Namely, inclusion criteria for the CAI group were: (1) having at least one significant ankle sprain, with the initial sprain occurring at least 12 months prior to study enrolment; (2) having sustained the most recent ankle sprain more than three months prior to enrolment; (3) having a history of ankle “giving way” or feeling of instability, which occurred at least two episodes in the last six months; and (4) reporting a score < 24 on the Cumberland Ankle Instability Tool (CAIT), < 90% on the Foot and Ankle Ability Measure (FAAM)–Activities of Daily Living (ADL) subscale and < 80% on the FAAM–Sport subscale. The Non-CAI group included healthy participants with no acute symptoms of an ankle injury and no history of ankle sprain or symptoms of instability. Exclusion criteria for both groups were: (1) having a history of previous surgeries to the musculoskeletal structures in either limb of the lower extremity; (2) having a history of fracture in either limb of the lower extremity requiring realignment; and (3) acute injury to musculoskeletal structures of other joints of the lower extremity in the previous three months. All athletes have right lower limb dominance. Also, only athletes with unilateral right ankle CAI were enrolled. Limb dominance was defined as the preferred limb to kicking a soccer ball^45^.

### Participants

Patients of the CAI group were matched on age and gender with individuals of the Non-CAI group. One hundred and twenty volleyball and basketball athletes were screened. For this study, we defined an athlete as a person having at least two training days/week and one match/week (in-season). The participants were classified into CAI and Non-CAI groups. Determination of the sample size was based on data from a pilot study that was calculated according to the inter-limb asymmetry of vGRF, a power analysis was conducted using G*Power version 3.1. The analysis revealed that given the effect size of 0.80, at least 21 participants per group was needed for a power of 0.80 and an alpha level of 0.05. Due to potential attritions, 56 athletes met the inclusion criteria (28 per group) and were recruited and tested for this study.

### Instrumentation

The vGRF data were collected at a sampling rate of 1000 Hz with Two force plates (Bertec Corp, 40 × 60, OH, USA) embedded into a 8-m walkway (approximately 4.5-m prior to force plate contact). Force plates were positioned respectively with 17 cm and 60 cm center to center distance in medial–lateral and anterior–posterior directions.

Electromyographic (EMG) data were recorded using EMG system (MT8 Model, MIE Medical Research, UK) applied over the tibialis anterior, peroneus longus, medial gastrocnemius, and gluteus medius. These muscles were selected because of their role in controlling the movement of the frontal and sagittal planes during walking. EMG signals were recorded at a sampling frequency of 1000 Hz and differential pre-amplified circuit (10^8^ Ω input impedance, 108 dB (typical) CMRR, 32 kHz bandwidth and gain of 4000 ×). Signals were measured using disposable electrodes (Australia's SKINTACT model) with a diameter of 10 mm. The EMG system was synchronized to the force plates.

### Procedure

First, the participants had to fill out the written informed consent form, CAIT^46^, FAAM-ADL, and FAAM-Sport^47^. Also, the participants reported the time since the last sprain and the total number of sustained ankle sprains. The steps of performing the tests and how to measure the variables were explained to the participants. Next, the muscles were prepared for EMG collection. After thorough shaving and cleaning of the skin with alcohol swabs, the electrodes were attached bilaterally over the muscles. All electrode placements were in accordance with SENIAM European protocols^48^.

The experimental section involved walking on an 8-m walkway. In order to approximate the test to normal conditions and prevent any possible change of gait pattern by focusing on walking speed, the participants should walk barefoot at their preferred speed. The participants performed three familiarization trials. Finally, they completed three trials with 30 s rest time between trials. Trials when the foot was not entirely on the force plates or the participants adapted their stride length or frequency in an attempt to hit the force plates were repeated.

### Data analysis

The vGRF data were filtered using a fourth-order, zero-phase lag, low-pass Butterworth filter with a cut-off frequency of 15 Hz^45^. The chosen threshold for determining the initial and final foot contact was operationalized as the time while vGRF exceeds 10 Newtons. Peak vGRF was measured per trial, and it was normalized relative to the participant's weight (%BW). For vGRF, two peaks were extracted: the maximum of the first half (first peak or impact peak) and the maximum of the last half (second peak) during the stance phase. Time to peak vGRF was defined as the time from initial heel contact to the first peak of vGRF^24^. The loading rate was defined as the rise in force (N/BW) from heel contact to the first peak of vGRF, divided by time to the first peak of vGRF^24^. These vGRF components were extracted for both limbs in both CAI and Non-CAI groups.

The speed of walking was measured as the length of step as divided by the duration of step, e.g., distance between the center of pressure (COP) anterior–posterior position at successive heel contact on each force plate (considering the 60 cm between them) divided by the time interval between those successive contacts on the force plates, took as the number of force samples divided by sampling frequency^45^.

The raw EMG data were filtered with a fourth-order, zero-phase lag, bandpass Butterworth filter with a cut-off frequency of 10–450 Hz^43^. In order to analyze the measured data, the stance phase of gait cycles was identified based on vGRF data (with a threshold set at 10 Newtons) and were normalized from 0 to 100%. Root mean square (RMS) activity index was used to calculate the amount of electromyography activity of the muscles during walking, during the contact phase (the first 25% of the stance phase), and the combined midstance/propulsion phase (the latest 75% of the stance phase). Analyses were performed on the RMS calculated with a moving window of 50 ms width. The RMS was normalized to the maximum voluntary isometric contraction (MVC) of each muscle and are reported as a percentage of the MVC (%MVC). The MVC was obtained during maximum contraction tests with manual muscle tests. The maximal value of MVC testing was collected over three trials for each muscle. A one-minute rest period was given between the MVC trials. All data processing occurred in MATLAB software (MathWork Inc., R2016a).

The inter-limb asymmetry for the variables of vGRF or muscles activity was calculated using an equation from previous studies^49,50^: Asymmetry Index = (injured (or dominant) limb − uninjured (or non-dominant) limb) × 2/ (injured (or dominant) limb + uninjured (or non-dominant) limb) × 100%. Therefore, a negative asymmetry would be indicative of a higher value for the uninjured (or non-dominant) limb. Asymmetry of less than 10% can be considered acceptable^49^.

### Statistical analysis

We used the Shapiro–Wilk test to determine the normality of the data (p < 0.05). Independent samples t-tests were computed to compare participant demographics, CAIT, FAAM-ADL, FAAM-Sport scores and the primary dependent variables between CAI and Non-CAI groups. In addition to inferential statistics, effect sizes (ES) (bias corrected Hedges’ g) and 95% confidence interval (CI) were also computed to estimate the precision and magnitude of group differences, given the multiple t-tests ran. ES were interpreted as weak (≤ 0.40), moderate (0.4 –0.69), or strong (≥ 0.70). Also, to compare the vGRF components and muscle activity between the CAI and Non-CAI groups, a two-way ANOVA was conducted to assess the interactions of group (CAI and Non-CAI) and limb (injured/dominant and uninjured/non-dominant). The injured and uninjured limbs of the CAI group were matched with the dominant and non-dominant limbs of the Non-CAI group, respectively. When significant differences were observed, pairwise post-hoc comparisons were performed using the Bonferroni test for multiple comparisons. All statistical analyses were computed using SPSS software, version 22 and statistical significance was considered when p < 0.05.

## Results

The demographic characteristics of the participants are summarized in Table 1. Results from the independent samples t-test revealed no significant differences between groups in age, height, mass, BMI, walking speed, and experience (P > 0.05). The CAI group reported lower CAIT, FAAM-ADL, and FAAM-Sport scores (P < 0.001) compared to the Non-CAI group (Table 1).

**Table 1:** Participant characteristics (Mean ± SD).

Characteristic	Group		P value
	CAI	Non-CAI	
Gender ratio (M/F)	19/9	19/9	
Age (years)	23.2 ± 2.9	24.3 ± 3.1	0.200
Height (cm)	173.7 ± 8.6	175.1 ± 7.7	0.525
Mass (kg)	68.5 ± 9.1	70.9 ± 9.5	0.328
BMI (kg/m^2^)	22.7 ± 2.7	23.2 ± 3.3	0.550
Walking speed (m/s)	1.22 ± 0.11	1.27 ± 0.13	0.110
Experience in sport competition (y)	5.3 ± 1.0	5.6 ± 1.2	0.274
CAIT score	16.8 ± 3.2	30.0 ± 0.0	< 0.001*
FAAM-ADL subscale	73.3 ± 7.1	100.0 ± 0.0	< 0.001*
FAAM-Sport subscale	64.9 ± 9.0	100.0 ± 0.0	< 0.001*
Time since last sprain ^a^ (y)	2.7 ± 2.0	0.0 ± 0.0	–
Previous sprains ^a^ (n)	5.4 ± 3.2	0.0 ± 0.0	–

*CAI* Chronic ankle instability, *CAIT* Cumberland ankle instability Tool, *FAAM-ADL* Foot and ankle ability measure-activities of daily.

*Denotes a significant difference.

^a^Indicates subjectively reported variable.

Patients with CAI demonstrated more asymmetry in the first peak of the vGRF (P < 0.001, ES = 1.38; CAI: 12.83 ± 9.67 vs Non-CAI: 1.41 ± 6.40), time to peak vGRF (P < 0.001, ES = 1.01; CAI: 14.77 ± 10.33 vs Non-CAI: 5.94 ± 6.42), and loading rate (P < 0.001, ES = 1.04; CAI: 13.95 ± 6.92 vs Non-CAI: 6.69 ± 6.73) (Table 2) compared to the Non-CAI group. There was no statistically significant difference in the asymmetry of the second peak of vGRF between groups (P > 0.05).

**Table 2:** Mean ± SD, effect size (ES) and 95% confidence interval (CI) for vGRF asymmetry between CAI and Non-CAI groups.

Asymmetry (%)	Group		ES (95%CI)
	CAI	Non-CAI	
First peak	12.83 ± 9.67	1.41 ± 6.40*	1.38 (0.80 to 1.97)
Second peak	− 2.26 ± 4.95	− 1.50 ± 4.90	− 0.15 (− 0.68 to 0.37)
Time to peak vGRF	14.77 ± 10.33	5.94 ± 6.42*	1.01 (0.46 to 1.58)
Loading rate	13.95 ± 6.92	6.69 ± 6.73*	1.04 (0.49 to 1.62)

*CAI* Chronic ankle instability, *vGRF* Vertical ground reaction force, *ES (95%CI)* effect size (95% confidence interval), *Indicates a significant difference from the CAI group (P ≤ 0.001).

During the contact phase of walking, patients with CAI demonstrated a greater inter-limb asymmetry of peroneus longus activity compared to the Non-CAI group (P = 0.003, ES = 0.82; CAI: 15.34 ± 11.40 vs Non-CAI: 7.29 ± 7.70) (Table 3). Also, during midstance/propulsion phase, the only statistically significant difference was in asymmetry of gluteus medius (P = 0.010, ES = 0.71; CAI: 14.15 ± 11.62 vs Non-CAI: 6.88 ± 8.34) (Table 3). There were no other significant group differences in asymmetry of muscle activity during the contact phase or midstance/propulsion phase of walking.

**Table 3:** Mean ± SD, effect size (ES) and 95% confidence interval (CI) for muscle activity asymmetry between CAI and Non-CAI groups.

Asymmetry (%)	Group		ES (95%CI)
	CAI	Non-CAI	
**Contact phase**			
Medial gastrocnemius	7.34 ± 4.96	4.77 ± 6.91	0.42 (− 0.10 to 0.95)
Tibialis anterior	− 3.28 ± 7.49	− 2.79 ± 5.28	− 0.07 (− 0.6 to 0.45)
Peroneus longus	15.34 ± 11.40	7.29 ± 7.70*	0.82 (0.27 to 1.37)
Gluteus medius	5.55 ± 6.90	4.61 ± 8.65	0.12 (− 0.40 to 0.64)
**Midstance/propulsion phase**			
Medial gastrocnemius	− 4.28 ± 6.94	− 3.20 ± 7.19	− 0.15 (− 0.67 to 0.37)
Tibialis anterior	− 7.61 ± 10.12	− 5.25 ± 8.64	− 0.25 (− 0.77 to 0.27)
Peroneus longus	7.48 ± 8.12	4.73 ± 6.20	0.37 (− 0.15 to 0.90)
Gluteus medius	14.15 ± 11.62	6.88 ± 8.34*	0.71 (0.17 to 1.25)

*CAI* Chronic ankle instability, *ES (95%CI)* effect size (95% confidence interval), *Indicates a significant difference from the CAI group (P ≤ 0.010).

**Figure 1:**
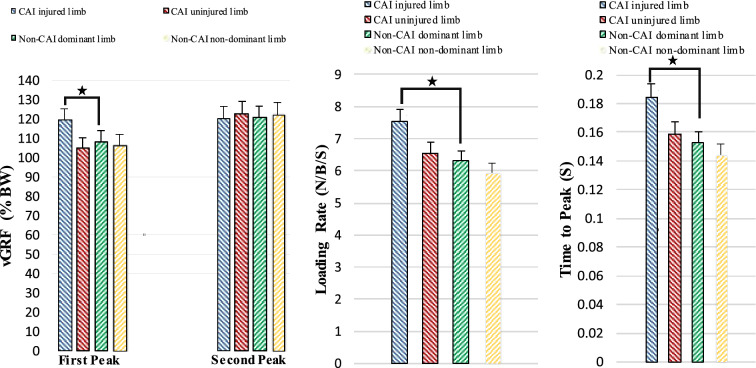
Average and standard division of vGRF, loading rate and time to peak in CAI and Non-CAI groups during walking. *CAI* Chronic ankle instability, *vGRF* Vertical ground reaction force. *Significant different between the limbs.

The two-way ANOVA showed significant group × limb interactions on the first peak of vGRF (F = 28.11, P < 0.001), time to peak force (F = 16.48, P < 0.001), and rate of loading (F = 24.68, P < 0.001). The post-hoc Bonferroni test showed significantly greater magnitude of the first peak of the vGRF (P < 0.001), time to peak vGRF (P = 0.002), and loading rate (P = 0.016) in the injured limb of patients with CAI compared to the dominant limb of the Non-CAI group (Fig. 1).

**Figure 2:**
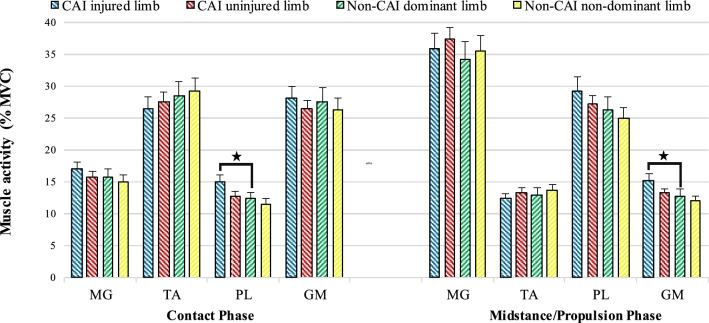
Average and standard division of muscles activity in CAI and Non-CAI groups during walking. *CAI* Chronic ankle instability, *MG* Medial gastrocnemius, *TA* Tibialis anterior, *PL* Peroneus longus, *GM* Gluteus medius. *Significant different between the limbs.

In addition, the ANOVA results showed significant group × limb interactions on the peroneus longus activity (contact phase) (F = 12.03, P = 0.001), and gluteus medius activity (midstance/propulsion phase) (F = 10.54, P = 0.002). The Bonferroni test showed significantly greater peroneus longus (P = 0.004) and gluteus medius activity (P = 0.011) for the injured limb of patients with CAI compared to the dominant limb of the Non-CAI group (Fig. 2). No other significant effects were observed.

## Discussion

This study aimed to compare the inter-limb asymmetry in vGRF and muscle activity between participants with and without CAI during walking. As hypothesized, patients with CAI presented greater inter-limb asymmetry in vGRF components and the activity of selected lower limb muscles than individuals in the Non-CAI group. Our main findings demonstrate differences in inter-limb asymmetry of the first peak of vGRF, time to peak vGRF, and loading rate between groups. Also, there were significant differences in inter-limb asymmetry of activity (at the contact phase) and gluteus medius activity (at the midstance/propulsion phase). The asymmetry observed in the vGRF components and muscles activity of the CAI group is due to changes in the injured limb. However, the results of this study did not show a significant difference between the uninjured limb of the CAI group and the non-dominant limb of the Non-CAI group in any of the variables.

Investigating inter-limb asymmetry can offer more comprehensive information to explain injured and uninjured limbs in the stance phase of walking. However, using the injured limb data alone may lead to unclear results, especially in athletes with CAI who may have to adapt to maintaining ankle stability. Also, our study tried to focus on unilateral right ankle CAI, gender, athlete performance, and the number of athletes in each sport to analyze a more homogeneous sample. Potentially, the most worrying long-term outcome of CAI is the development of post-traumatic osteoarthritis^2^. Therefore, preventive initiatives are necessary to reduce the long-term consequences of these injuries^51^, and the first step towards progress in this area is to examine the walking features of these patients.

Patients with CAI demonstrated inter-limb asymmetry in the first peak of vGRF, time to peak vGRF and loading rate compared to the Non-CAI group. The primary finding is that patients with CAI in the injured limb had a significantly higher first peak of vGRF and loading rates and significantly less time to peak vGRF than the non-dominant limb of the Non-CAI group, consistent with broader biomechanical literature on CAI^13,23,24,52^. Previous studies confirm that patients with CAI experience different magnitudes of vGRF compared to the Non-CAI group during running^23^, jump landing^52^, and cutting^53^. However, few studies reported vGRF data in patients with CAI during walking^13,24,25,41^. Read et al.^25^ and Son et al.^13^ noted increased vGRF in patients with CAI than the Non-CAI group during early stance of walking. Contrary to our findings, Moisan et al. in their published abstract, reported no significant differences in vGRF between individuals in the CAI and Non-CAI groups^41^. Moreover, Wikstrom et al.^24^ reported that patients with CAI had higher loading rates and less time to peak vGRF compared to the Non-CAI group during walking. However, they reported no differences between groups in magnitude of vGRF during overground walking at a self-selected pace^24^. This difference between Wikstrom et al. and our study is due to the point that they normalized the vGRF to body weight and gait velocity, while in our study it was only body weight.

Patients with CAI may compensate for ankle instability with a more rigid walking strategy as a mechanism to protect the ankle joint from recurrent sprains. For example, patients with CAI have decreased dorsiflexion angle or increased plantar flexion^13,18,32^, increased plantar flexion moment^13^ and increased loading rates at initial contact^24^ in comparison to the Non-CAI group. Less dorsiflexion or more plantar flexion during the early stance of walking may reduce the ability to absorb impact at heel strike. Cumulatively, these findings would suggest that patients with CAI have a limited ability to appropriately modulate vGRF during walking. The primary analyses also show that the CAI cohort has problems in dampening forces during gait termination^54^, indicating limitations in force modulation across gait-related activities.

Although the increased vertical forces in the present study are considered to be the underlying causes of musculoskeletal stress injuries and overuse injuries^52^, the present results are likely to be a neuromuscular response that may be attributed to sensorimotor impairments that increases ankle joint stability during early stance as a protective mechanism. However, they may contribute to post-traumatic ankle osteoarthritis. Patients with post-traumatic ankle osteoarthritis also show changes in peak force and loading rates during dynamic activities such as stair climbing^55^ and walking^56^. Additionally, it has been shown that articular cartilage catabolism changes relative to alterations in BW and GRF because of walking^57^. We speculate that these data points can be linked by extrapolating the increased force across the number of steps per day over years. Therefore, changes observed in the loading pattern may increase wear on the talar cartilage and aid in the progression of post-traumatic osteoarthritis. This theoretical model is supported by previous research in patients with other musculoskeletal injuries. For example, increased vGRF was associated with biochemical markers of cartilage degradation in patients with anterior cruciate ligament reconstruction (ACLR)^58,59^. Despite preliminary evidence from related studies in ACLR literature, the relationship between cartilage degradation and increased peak force and loading rates during walking has not been explored in CAI populations. However, only a prospective research design will be able to test this hypothesis adequately.

This study showed significantly greater peroneus longus asymmetry in the contact phase that did not continue into the midstance/propulsion phase participants in the CAI versus the Non-CAI groups. Also, our findings show that patients with CAI in the injured limb had significantly higher peroneus longus activity relative to the dominant limb of the Non-CAI group. This result agrees with previous investigations demonstrating greater activity of the peroneus longus muscle during early stance^17,18^ in patients with CAI. However, the results from our muscle activation during walking should be interpreted cautiously. For example, our results are not in line with researchers who reported a decrease in peroneus longus during the initial stance^13,60^, or lack of difference between the groups^20,21,42,43^. The variations in the reference task adopted for EMG normalization by different researchers induce a problematic comparison of results across studies. For example, the reference task was MVC in this study, while others used squatting positions^13^ or quiet standing^60^. Thus, knowing if the groups utilized more or less motor activity while doing those normalization tasks represents a priority for future research.

The higher activation of the peroneus longus could be a compensatory strategy to avoid ankle inversion movements leading to ankle sprains, since a number of studies reported an increased ankle inversion^13,18^ and plantar flexion^13^, as well as an increased lateral COP displacement^17,60^ during walking in patients with CAI compared to the Non-CAI group. These differences are risk factors for lateral ankle sprains. In addition, it is likely that the abnormal peroneus longus activation is a strategy to compensate for neural alterations affecting ankle evertor muscles^61^. This increased duration and magnitude of peroneus longus activity in patients with CAI contrasts that of healthy individuals who activate the peroneus longus during midstance to help initiate pronation and stabilize their first ray during propulsion phases of gait^62^.

In the midstance/propulsion phase, the inter-limb asymmetry in the gluteus medius activity was significantly different between individuals in the CAI and Non-CAI groups. Also, our results indicate that patients with CAI in the injured limb had significantly higher gluteus medius activity than the dominant limb of the Non-CAI group. Modified movement control patterns in the muscles of the proximal joints have been identified in patients with CAI^16,63^. For example, Koldenhoven et al. reported that the gluteus medius muscle had higher activity before heel contact, during the second half of the stance phase, and the first quarter of the swing phase as compared to healthy participants^42^. They suggested that Proximal changes in gluteus medius muscle activity may represent a coping mechanism that CAI patients utilize to generate a wider base of support or stabilize their lower limb during walking^42^. Although Feger et al. did not find significant differences in EMG amplitude and onset of gluteus medius activity during walking, they reported earlier onset activity as well as overactivity just before and just after initial contact^20^. Contrary to our findings, Moisan et al.^43^ and Son et al.^13^ observed decreased gluteus medius activity during the stance phase. These differences in results may be due to the use of different methods in EMG normalization, as they used the peak RMS amplitude while walking^43^ and the squat position^13^ for normalization. Also, in the study of Moisan et al.^43^, participants used shoes while walking, and the whole stance phase was considered, while our participants were barefoot and we considered several stance phases. Accordingly, future studies are needed to investigate the gluteus medius muscle function in patients with CAI during walking.

The gluteus medius muscle contributes to balance control of the upper extremity (e.g., pelvis, trunk, upper extremities, and head) in the frontal plane while the early stance and midstance phases of walking^64^. It has been argued that total-body balance in the frontal plane while walking is modulated by the subtalar and hip joints that control the center of mass position and upper extremity motion^64^; thus, the ankle and hip function synergically. When the foot enters a susceptible position in danger of lateral ankle sprain (e.g., greater inversion^13,18,32^) in the frontal plane, wrong foot placement could be rectified by an interface between hip abductors and evertors, which shows the significance of gluteus medius function in CAI. Given that increased gluteus medius activation would decrease hip adduction (e.g., lateral pelvic tilt) and create efficient balance of the upper extremity, which impedes lateral movement of center-of-mass, potentially stops patients with CAI to be inflicted by lateral ankle sprains. So, increased gluteus medius activation can be considered as a “safety strategy” to compensate for a changed compensatory motor control system. Continued increased functional load on the hip joint can increase the ground reaction shear forces.

Asymmetry, occurring due to increased muscle activity and vGRF components in the injured limb in the CAI group, is a compensatory (coping) gait pattern that may allow patients to increase limb stability. The asymmetry created in the CAI group indicates altered gait mechanics, which could increase loads on lower extremity joints in the injured limb, thus potentially increasing the risk of re-injury^25^. Increased abnormal stress on the osseous structures and soft tissues can cause osteoarthritis prematurely^56^. The collective aberrant kinetic and muscle activation patterns demonstrated by patients with CAI in this study suggest that therapeutic interventions should focus on restoring normal neuromuscular function during walking. Specifically, targeted interventions to restore vGRF symmetry, reduce peroneus longus asymmetry, and restore gluteus medius function may have important implications for the progression from CAI to ankle joint degeneration. Real-time biofeedback tools and injury prevention programs can modify vGRF^65,66^. Although there is no evidence-based recommendation for CAI gait training, it may support the rehabilitation of inter-limb asymmetry and reduce the risk of re-injury^67^. We surmise that for asymmetry changes to be maintained by patients with CAI, a more inclusive approach to gait training is probably needed to restore proper vGRF and improve the patterns of muscle activity as these factors play a role in an patient’s loading pattern.

The present study had several limitations that should be mentioned. First, although the combination of vGRF and EMG was used in this study to evaluate the difference in asymmetry between the groups, kinematic evaluation of joint movements, which could contribute to the comprehensiveness of the study, was not performed in this study. Therefore, the interpretation of our results should be considered with some caution. Future research is needed to simultaneously examine EMG, vGRF and lower limb kinematics while walking to better understand the implications of our findings. Second, we compared two discrete time points throughout the gait cycle in our study. Although this analysis is recognized internationally^18–20,22,42^, other gait biomechanics studies on patients with CAI adopted functional statistics (e.g., statistical parametric mapping; SPM or functional linear models) to compare all data points across the gait cycle^13,68,69^, thus offering opportunities for future research in this field. Third, considering that participants in this study had a mean age of 23 years, our results can be generalized to only the targeted populations. Fourth, given the cross-sectional nature of this study, it is unclear whether the current CAI leads to the observed alterations or if gait asymmetry facilitated the development of CAI. Lastly, it is possible that the asymmetry is already present before the injury acting as a risk factor for injury/instability. The design of our research (cross-sectional study) prevent us from any definitive conclusion. Thus, future studies should investigate the topic using a prospective design (e.g., longitudinal).

## Conclusions

Patients with CAI walk with greater inter-limb asymmetry in muscle activity and vGRF in different phases of the gait cycle compared to the Non-CAI group. These motor behaviour changes could have occurred to maintain joint stability in patients with CAI. Our results could inform future studies on gait training aimed to reduce asymmetry during walking in patients with CAI.
